# Grand Challenges in Microalgae Domestication

**DOI:** 10.3389/fpls.2021.764573

**Published:** 2021-09-23

**Authors:** Eric Maréchal

**Affiliations:** Laboratoire de Physiologie Cellulaire et Végétale, CNRS, CEA, INRAE, Université Grenoble Alpes, IRIG, CEA Grenoble, Grenoble, France

**Keywords:** microalgae, domestication, algae based bioproducts, lipids, pigments, biofuel, green chemistry

## Introduction

The term “microalgae” is a practical designation for photosynthetic unicellular cells, embracing organisms from two domains of life, i.e., Bacteria (Cyanobacteria) and various clades of Eukaryota deriving from primary (Archaeplastida) or secondary (e.g., Stramenopile) endosymbiosis events. In spite of this dispersed taxonomic distribution, microalgae share features that make them somehow “alike”. Oxygenic photosynthesis derives from a common origin and makes microalgae prominent in trophic networks, as primary producers. They are unicellular or form very small colonies, and their cultivation rely on common methods, with provision of light, CO_2_, water, and nutrients. Microalgae produce valuable molecules, like glycans, lipids, pigments, proteins, etc. Thus, although inappropriate in botanical or taxonomic sense, the term “microalgae” takes its legitimate meaning in ecology and human industry. This is both a weakness, when trying to transfer knowledge from one organism to another, and a strength, when addressing similar biotechnological questions. The development of a microalgae-based industry has become a societal challenge in the past decade. The climatic emergency and pressure on arable lands make the need for novel carbon-free and sustainable productions each day more urgent. Applications range from food, health, green chemistry to biofuels, with the promise of biomolecules produced from CO_2_ captured from the atmosphere or carbon-emitting industries. In this context, an “algae sector” has emerged, gathering actors specialized in algae cultivation, harvesting, extraction processes, and biorefinery.

Turning a wild algal strain into an “algal crop”, i.e., “domesticating” a microalga, represents a grail, because initial traits of interest may be present, like a relatively high level of oil, carbohydrates, pigments, etc., but the path to an enhanced, reproducible and up-scalable yield is extremely challenging.

Some lessons can be learnt from agriculture and give a novel stimulus to research in the microalgae sector. When one walks in nature, does he or she find wild plants resembling wheat, maize, tomato, sunflower, rapeseed, etc? Crop plants look giant and obese, when compared to their wild counterparts. Furthermore, after harvest, it is rare that cultivated seeds escape and invade uncultivated areas. Plant domestication is therefore focused on productivity and quality, but not on fitness in competition with wild communities. The strong difference between wild and domesticated plants illustrates that gains in yield should be obtainable in other branches of life, keeping in mind that cultivated plants are diploid, whereas the majority of currently cultivated microalgae are haploid.

## Basic Lessons from Agriculture

The domestication of crop plants started during the Neolithic Period and can be reconstructed based on population genetics studies, the analysis of archeological artifacts and the experimental validation of reasonable hypotheses. In this first period of agriculture, the purification of strains by selecting traits of interest and the control of breeding techniques were consciously or unconsciously the strategies of choice.

If we focus on “gene targets” governing traits of interest, a well-known example is the domestication of teosinte into the crop maize. It involved “domestication genes” controlling the growth of lateral stems or formation of naked kernels (Chen et al., 2021). With genetic and agronomic strategies developed in the last century, additional mutations allowed the cultivation of maize virtually in all places in the world, with remarkably high yields. Targeting genes can now be much faster using CRISPR/Cas9-based methods (Lemmon et al., 2018). It is also feasible to produce plants devoid of any foreign DNA by methods collectively called next-generation or new breeding techniques (NBTs) (Holme et al., 2019). Some of the NBTs target genes with a Cas9-coding DNA vector who does not integrate into the plant genome, or even a microinjected Cas9 protein and gRNAs, whereas other NBTs are based on non-targeted random mutations (Holme et al., 2019; Anders et al., 2021). The concept of “gene target” proved determinant in the understanding of some domesticated organisms. The “*de novo*” domestication of tomato could thus be reconstructed experimentally gene-by-gene (Lemmon et al., 2018). This being stated, when transferred to microalgae, it would be naïve to limit the understanding of domestication to the modification of well-selected genes. Some traits depend on complex interactions, and strategies should also consider regulatory genes controlling broad metabolic, physiological, or developmental processes, combinations of genes and non-targeted multiple random mutations.

Plant domestication also highlights the benefits of features, which cannot be reduced to a gene target approach. In schematic terms, improved traits can emerge from specific “rearrangements” or “duplications of chromosomes”. In the simplest example in maize, the crossing of homozygous parent lines can give rise to an heterozygous “hybrid” with superior robustness and productivity, a phenomenon called “heterosis” (Srivastava et al., 2020; Chen et al., 2021). Polyploidy can also “improve” patterns of gene expression (Schaart et al., 2021). On the one hand, it can be a high level of “autopolyploidy”, which can be induced by a variety of techniques (Chen et al., 2020). On the other hand, “allopolyploids” deriving from the crossing of very close but distinct species, can give rise to progenies retaining the parent chromosomes and exhibiting higher productivity and robustness. Rapeseed (*Brassica napus*) is an allotetraploid, containing chromosomes from *B. rapa* and *B. olearacea* parents (Mason and Snowdon, 2016). Likewise, common wheat (*Triticum aestivun*) is a hexaploid combining chromosomes from diverse *Triticum* and *Aegilops* species (Parisod and Badaeva, 2020). The improvement of algal strains by developing hybrids, autopolyploids or allopolyploids is currently barely explored.

The last lesson from plant domestication may be that growth, productivity, resistance to diseases, etc., can be addressed by domestication strategies, but the response to abiotic environmental stressors, like drought or nutrient scarcity are more difficult questions (Li et al., 2021).

## Challenges in Microalgae Domestication

With the notable exceptions of yeast and some fungi, or *Chlamydomonas*, genetics is poorly developed in microbiology. Due to their unicellular nature, microalgae populate environments (oceans, rivers, soils, snow, ice, extreme habitats, etc.) primarily by mitotic divisions, either in haploid or diploid forms. The majority of microbial organisms are not cultivable (Ding et al., 2014). Strains collected in nature can be purified using a cell sorter or by serial cultivation on petri dishes until obtaining clonal axenic lines. Sometimes it is not possible to separate a microalga from its companion bacteria (Lupette et al., 2016). Only in response to environmental or physiological triggers, do gametogenesis, sexual reproduction and meiosis occur (Lopez et al., 2015). Whereas, breeding and shuffling of natural alleles are major drivers of diversity in plants and animals, they do not seem to play this role in microalgae. Genetic diversity relies on genomic mutation rates and transfers of genetic material within and between species. A bias in the microbiological approach of domestication, compared to agriculture, is that the majority of strategies aims at developing an improved clonal strain, and disregard more global approaches with multiple series of mutant lines, which could be crossed, combined and improved.

A first challenge is to pursue the exploration of biodiversity. This includes efforts to resolve the question of non-cultivable strains. Some qualitative traits like the capacity to produce a biomolecule of interest may serve as a starting point for domestication attempts; nevertheless, the capacity to grow fast and produce biomass should be considered an important initial property for further consideration.

A second challenge is the lack of sequenced genomes and the difficulty to genetically transform many non-model and emerging model species. Let us cover the biodiversity of microalgae pointing to some examples of current efforts on “algal crop” models.

-In Cyanobacteria, spirulina (*Arthrospira platensis*) is “technically” haploid. It contains a rich equipment of restriction enzymes, and methods to efficiently transform this popular Cyanobacterial crop have been made available only recently (Jeamton et al., 2017; Dehghani et al., 2018).
-In Eukaryota, the Archaeplastida comprise three lineages: Green Algae, Red Algae, and Glaucophyta. The Green Alga genetic model is *Chlamydomonas reinhardtii*, with a genome sequenced two decades ago (Blaby et al., 2014; Lopez et al., 2015). Its life cycle relies mainly on haploid asexual divisions; sexual reproduction can be controlled *in vitro* (Wilson, 2008). Nuclear and chloroplastic transformations are possible and multiple methods have been developed for gene editing (Ghribi et al., 2020). It is thus considered as a model for synthetic biology (Scaife et al., 2015). In spite of these advantages, its limited biomass and productivity does not make *C. reinhardtii* a real crop (Butler et al., 2020). *Chlorella* and *Dunaliella* species have life cycles close to that of *C. reinhardtii*. *Chlorella* are maintained in haploid form, and it is difficult to know whether sexual reproduction could be obtained. Genomic data have been made available recently for a few strains (Wu et al., 2019) and transformation mediated by *Agrobacterium tumefaciens* is possible (Cha et al., 2012; Sharma et al., 2021). *Dunalliella* has a main haploid cycle, but sexual reproduction is known. Draft genomic data are available (Polle et al., 2017) and nuclear and chloroplastic transformations have been validated recently (Dehghani et al., 2017; Norzagaray-Valenzuela et al., 2018; Bahador et al., 2019; Song et al., 2019). Technically both *Chlorella* and *Dunaliella* could be amenable to intensive genetic engineering. *Scenedesmus* and *Haematococcus* species are Green Algae producing pluri-nuclear cells, with four to eight haploid nuclei, in the course of cell fission. Genomes of *S. obliquus* (Nag Dasgupta et al., 2018; Astafyeva et al., 2020) and *H. pluvialis* (Luo et al., 2019; Morimoto et al., 2020) are available. Transformation of *S. obliquus* via *A. tumefasciens* is feasible (Suttangkakul et al., 2019). Both nuclear and chloroplastic transformations have been described for *H. pluvialis* (Yuan et al., 2019; Wang et al., 2020; Cui et al., 2021). Finally, Charophyta are Green Algae close to Embryophyta, propagating by haploid asexual division; sexual reproduction is known. Genomic data are available for the Charophyta model *Klebsormidium* (Hori et al., 2014), and transformation and gene editing have been obtained in a *Closterium* species (Abe et al., 2011). In Red Algae, *Galdieria sulphuraria* is considered for its cultivation at high temperature and acidity. Its small genome has been known for nearly two decades (Barbier et al., 2005), and genetic engineering is possible (Fujiwara et al., 2019).
-In photosynthetic Stramenopiles, a branch of Eukaryota deriving from a secondary endosymbiosis, the diatom *Phaeodactylum* and the eustigmatophytes *Nannochloropsis/Microchloropsis* species are crop models studied by multiple groups worldwide. Robust genomic data (Bowler et al., 2008; Vieler et al., 2012) and tools for genetic engineering are available; multiple examples show the power of gene editing to improve traits and domesticate these lines (Siaut et al., 2007; De Riso et al., 2009; Kilian et al., 2011; Cao et al., 2012; Daboussi et al., 2014; Dolch et al., 2017; Poliner et al., 2018a; Nobusawa et al., 2019; Billey et al., 2021). Remarkably, whereas eustigmatophytes are maintained in haploid form, the vegetative cells of diatoms are diploid.

In the above listed microalgae, strategies developed on multiple gene targets rely on recent technological developments. Progresses in non-GMO plant domestication also inspire the search for methods allowing the transient expression of Cas9 and gRNAs, for instance, by an episomal DNA, removed after lifting the vector selection pressure, to generate strains without any foreign DNA (Poliner et al., 2018b; Sharma et al., 2018; Moosburner et al., 2020).

A third challenge is to control meiosis, gametogenesis, and sexual reproduction. Conventional genetics could boost targeted strategies, by allowing allelic rearrangements. Major breakthroughs could be anticipated if sexual reproduction could be controlled in routine in *Chlorella, Dunaliella, Scenedesmus, Klebsormidium, Phaeodactylum* etc. We need to keep in mind that in some algal species, the existence of mating types may limit the capacity to develop homozygous diploids requiring the development of self-mating lines (Kariyawasam et al., 2019).

A fourth challenge lies in the development of systems allowing the control of chromosomic combinations and duplication. Heterosis relies directly on the capacity to obtain heterozygous hybrids from homozygous lines. Diatoms have diploid vegetative cells allowing the exploration of this property. The development of autopolyploids from haploid cells has been attempted in pioneering experiments by treatments of *Chlamydomonas* with molecules blocking cell division, like colchicine (Wetherell and Krauss, 1956) or colcemide (Kwak et al., 2017; Kariyawasam et al., 2019). In diploid *Chlamydomonas* lines, the lipid yield was improved (Kwak et al., 2017). Concerning allopolyploidy, sexual reproduction between distinct parental species needs to occur. The genome of the diatom *Fistullifera solaris* derives from two distinct parental species (Tanaka et al., 2015). *F. solaris* is oleaginous, suggesting that allopolyploidy may have a positive impact on productivity in diatoms as well. Future work on cell fusion may help circumventing the need for a sexual reproduction, within or between species, to further explore this potential.

Eventually, a last challenge concerns random mutagenesis and *in vivo* directed evolution approaches (Crook et al., 2016) as a way to domesticate microalgae (Pourmir and Johannes, 2012; Rossoni and Weber, 2019). Such strategies require excellent screening methods for selection of improved lines. They also need sequencing efforts to characterize mutants and comprehend the relation between genomic changes and gained properties.

In conclusion, microalgae domestication is in its infancy. The exploration of biodiversity needs to be pursued. Given the development of enabling methods in an increasing number of microalgal groups, there is no doubt that the coming decade will be marked by fascinating results.

## Author Contributions

EM has conceived and written the manuscript.

## Funding

EM was supported by the Agence Nationale de la Recherche (Alpalga ANR-20-CE02-0020, Oceanomics ANR-11-BTBR-0008, GlycoAlps ANR-15-IDEX-02, GRAL Labex ANR-10-LABEX-04, and EUR CBS ANR-17-EURE-0003) and Institut Carnot3BCAR.

## Conflict of Interest

The author declares that the research was conducted in the absence of any commercial or financial relationships that could be construed as a potential conflict of interest.

## Publisher's Note

All claims expressed in this article are solely those of the authors and do not necessarily represent those of their affiliated organizations, or those of the publisher, the editors and the reviewers. Any product that may be evaluated in this article, or claim that may be made by its manufacturer, is not guaranteed or endorsed by the publisher.
